# Extending Stochastic Network Calculus to Loss Analysis

**DOI:** 10.1155/2013/918565

**Published:** 2013-10-20

**Authors:** Chao Luo, Li Yu, Jun Zheng

**Affiliations:** National Laboratory for Optoelectronics, Huazhong University of Science and Technology, Wuhan 430074, China

## Abstract

Loss is an important parameter of Quality of Service (QoS). Though stochastic network calculus is a very useful tool for performance evaluation of computer networks, existing studies on stochastic service guarantees mainly focused on the delay and backlog. Some efforts have been made to analyse loss by deterministic network calculus, but there are few results to extend stochastic network calculus for loss analysis. In this paper, we introduce a new parameter named loss factor into stochastic network calculus and then derive the loss bound through the existing arrival curve and service curve via this parameter. We then prove that our result is suitable for the networks with multiple input flows. Simulations show the impact of buffer size, arrival traffic, and service on the loss factor.

## 1. Introduction

Loss is one of the key indicators of QoS. Traditional methods for loss analysis aim at estimating the work load loss ratio based on the approximations of buffer overflow probability [1–4]. However, there are two main drawbacks in these methods. First, it is difficult to calculate the buffer overflow probability for some input processes. Second, the relation between loss ratio and buffer overflow probability is often hardly quantifiable [1].

Network calculus is a theoretical framework for analysing performance guarantees in computer networks [5]. The deterministic network calculus, firstly proposed by Cruz [6, 7], can be used to obtain the delay bound and backlog bound in the worst case [8]. Some efforts have been made to apply deterministic network calculus to get the loss probability or loss bound. In [9], a new composable service model with loss is proposed, but it can only be used for a specific scheduling algorithm mentioned in the paper, which limits the scope of its application. A loss bound is derived with envelop and moment generating function in [10]. The indirect method for packet loss approximation mentioned in [11] is also based on deterministic arrival curve and deterministic service curve. However, the deterministic network calculus usually results in overly pessimistic performance bounds that are rarely attained which leads to low utilization of network resources. This defect limits the range of its application. Consequently, loss analysis based on deterministic network calculus is not suitable for many applications.

As a probabilistic extension of the deterministic network calculus, stochastic network calculus has been studied by some researchers [12, 13]. Stochastic network calculus uses some stochastic arrival curves and some stochastic service curves to characterize the arrival process and the service process, which can provide stochastic QoS guarantees [14]. This feature makes stochastic network calculus suitable for many applications to which deterministic network calculus can-not be applied. Hence, it is very significative to find a way to analyse loss by using stochastic network calculus.

In this paper, we do not assume that the network is lossless but consider a network with finite buffer size which is not large enough to avoid loss occurring. Accounting for the nature of stochastic arrival curve and stochastic service curve, it is very difficult to calculate the amount of packets that have been dropped by directly using stochastic network calculus. To fill this vacancy, we propose a novel method to calculate the loss bound by using stochastic network calculus. We introduce a new parameter, named loss factor, into stochastic network calculus. Via this new parameter, we establish a loss analysis model based on traffic-amount-centric stochastic arrival curve and stochastic strict service curve.

The rest of this paper is organized as follows. The notations and the theoretical background of stochastic network calculus are introduced in Section 2. In Section 3, we present and prove our loss analysis model. In Section 4, we show that our analysis model can be also applied to the scenario with multiple input flows. We explore the relationships between the loss factor and the buffer size, the loss factor and the arrival, and the loss factor and the service by simulation in Section 5. In Section 6, we make a brief conclusion.

## 2. Background

In this section, we introduce the notations and some concepts of stochastic network calculus which will be used in this paper.

### 2.1. Notations

We use *A*(*t*), *S*(*t*), A*(*t*), *B*(*t*), and *L*(*t*) denoting the arrival process, the service process, the departure process, the backlog at time *t*, and the loss process in time interval (0, *t*], respectively. We also denote *A*(*s*, *t*) ≡ *A*(*t*) − *A*(*s*), *S*(*s*, *t*) ≡ *S*(*t*) − *S*(*s*), A*(*s*, *t*) ≡ A*(*t*) − A*(*s*), *B*(*t*) = *A*(*t*) − A*(*t*) and *L*(*s*, *t*) ≡ *L*(*t*) − *L*(*s*). Conventionally, we assume that *A*(*t*), *S*(*t*), A*(*t*), *B*(*t*), and *L*(*t*) have zero value at *t* = 0.

We denote the set of nonnegative wide-sense increasing function by *ℱ*, where for each function *f*(·), there holds

(1) $$\begin{array}{c} ℱ=\left\{f\left(\cdot \right):\forall 0\leq x\leq y,0\leq f\left(x\right)\leq f\left(y\right)\right\}. \end{array}$$

We denote the set of nonnegative wide-sense decreasing function by $\bar{ℱ}$, where for each function *f*(·), there holds

(2) $$\begin{array}{c} \bar{ℱ}=\left\{f\left(\cdot \right):\forall 0\leq x\leq y,0\leq f\left(y\right)\leq f\left(x\right)\right\}. \end{array}$$

### 2.2. Stochastic Network Calculus

Min-plus algebra has been wildly used in network calculus. For given functions *f* and *g*, the min-plus convolution operator ⊗ and the min-plus de-convolution ⊘ are defined by

(3) $$\begin{array}{c} \left(f⊗g\right)\left(t\right)=\underset{0\leq s\leq t}{\inf}\left(f\left(s\right)+g\left(t-s\right)\right),\\ \left(f⊘g\right)\left(t\right)=\underset{t\geq 0}{\sup}\left(f\left(t+s\right)-g\left(s\right)\right). \end{array}$$

The following properties of ⊗ will be used in the latter paper [5]. Let $f,g,h\in \bar{ℱ}$:

- (closure of ⊗) $f⊗g\in \bar{ℱ}$;
- (associativity of ⊗) (*f* ⊗ *g*) ⊗ *h* = *f* ⊗ (*g* ⊗ *h*);
- (commutativity of ⊗) *f* ⊗ *g* = *g* ⊗ *f*.

Many kinds of stochastic arrival curves and stochastic service curves are proposed in stochastic network calculus. We use the traffic-amount-centric stochastic arrival curve and the stochastic strict service curve in our loss analysis model, and their definitions are as follows.

**Definition 1 (t.a.c. stochastic arrival curve [12])**

A flow is said to have a traffic-amount-centric (t.a.c.) stochastic arrival curve *α* ∈ *ℱ* with bounding function $f\in \bar{ℱ}$, denoted by *A* ∼_ta_〈*f*, *α*〉; if for all 0 ≤ *s* ≤ *t* and all *x* ≥ 0, there holds

(4) $$\begin{array}{c} P\left\{A\left(s,t\right)-\alpha \left(t-s\right)>x\right\}\leq f\left(x\right). \end{array}$$

**Definition 2 (stochastic strict service curve [12])**

A system is said to be a stochastic strict server providing stochastic strict service curve *β*(*t*) with bounding function $g(x)\in \bar{ℱ}$, denoted by *S* ∼_ssc_〈*g*, *β*〉, if during any period (*s*, *t*], the amount of service *S*(*s*, *t*) provided by the system satisfies

(5) $$\begin{array}{c} P\left\{S\left(s,t\right)<\beta \left(t-s\right)-x\right\}\leq g\left(x\right) \end{array}$$

for any *x* ≥ 0.

### 2.3. Related Work

As far as we know, there are very few results on loss analysis in the context of stochastic network calculus. In [15, 16], the authors proposed a new stochastic network calculus for loss analysis. However, there are two main limitations in their results. First, the arrival curve and the service curve they proposed are not suitable for the arrival traffic and the service that have high burstiness. Second, the upper bound of the loss ratio can be derived only under the condition that the system has a certain buffer size *b* which is determined by the arrival curve and the service curve. That means if the buffer of a system is not equal to *b*, the bound of the loss ratio can-not be obtained.

Existing results on loss, including the one mentioned in the previous paragraph, are obtained approximately from the backlog analysis. However, this approximation in general gives loose bounds [17]. Assume that the buffer size is *b* and the approximate upper bound of the loss probability is *P*{*B*(*t*) > *b*}. The main problem in the approximation is that the buffer size is assumed to be infinite in stochastic network calculus; that is to say the system is lossless. But in the real system, the backlog can never exceed *b*, because the exceeded packets are dropped. This is the reason why the bounds are loose. It is hence of great importance to study the loss directly, not by the approximation of the backlog.

## 3. Loss Bound Based on Stochastic Network Calculus

Unlike delay and backlog, it is not easy to directly use some stochastic arrival curves and some stochastic service curves to denote loss within existing framework of stochastic network calculus. In this section, we firstly give the definition of loss period and then present the loss bound based on stochastic network calculus.

**Definition 3 (loss period)**

In a network system, a duration is called a loss period if it begins when the buffer is full and the arrival rate is larger than the service rate, and it ends once the arrival rate is smaller than the service rate.

If (*s*, *t*] is a loss period, then the amount of loss during (*s*, *t*] is *A*(*s*, *t*) − *S*(*s*, *t*). Then the loss bound *L*(*s*, *t*) can be expressed as

(6) $$\begin{array}{c} P\left\{L\left(s,t\right)>x\right\}=P\left\{A\left(s,t\right)-S\left(s,t\right)>x\right\}. \end{array}$$

In a period of time, the occurrences of loss periods are difficult to confirm. Hence, the result provided by Lemma 4 [12] below is indispensable to our following analysis.

**Lemma 4**

For any random variables *X* and *Y*, and ∀*x* ≥ 0, if *P*{*X* > *x*} ≤ *f*(*x*) and *P*{*Y* > *x*} ≤ *g*(*x*), where $f,g\in \bar{ℱ}$, then

(7) $$\begin{array}{c} P\left\{X+Y>x\right\}\leq \left(f⊗g\right)\left(x\right). \end{array}$$

The result provided by Lemma 4 can be easily extended to *n* (*n* > 2) variables.

In essence, the size of the buffer of a system definitely has impact on the loss. In the current theoretic framework of stochastic network calculus; however, the buffer size is usually assumed to be infinite and the impact of the buffer size can-not be embodied. Hence, we introduce a new parameter called loss factor into stochastic network calculus.

**Definition 5 (loss factor)**

In stochastic network calculus, loss factor is a parameter which indicates the combined impact of arrival, service, and buffer size on loss.

**Figure 1:**
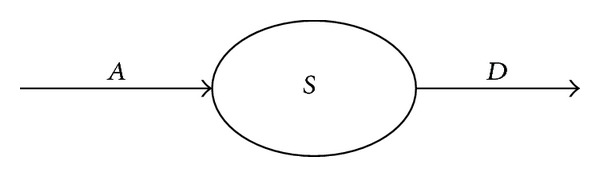
A simple network.

We consider a simple network with one input flow as depicted in Figure 1; then, we present the loss bound in Theorem 6 below.

**Theorem 6**

Consider that a flow arrives into a network system with finite buffer. If the flow has a t.a.c. stochastic arrival curve *α* denoted by *A* ∼_*ta*_〈*f*, *α*〉 and the system provides a stochastic strict service curve *β* denoted by *S*∼_*ssc*_〈*g*, *β*〉, then there exists a *r*, where *r* > 1 for all *x* ≥ 0 and 0 ≤ *s* ≤ *t*, such that

(8) $$\begin{array}{c} P\left\{L\left(s,t\right)>x\right\}\leq r\left(f⊗g\right)\left(x+\beta \left(t-s\right)-\alpha \left(t-s\right)\right). \end{array}$$

**Proof**

During time [*s*, *t*], we assume that there are *n* loss periods [*s*_1_, *t*_1_], [*s*_2_, *t*_2_],…, [*s*_*n*_, *t*_*n*_](*s* ≤ *s*_1_ ≤ *t*_1_ ≤ *s*_2_ ≤ *t*_2_ ≤ ⋯≤*s*_*n*_ ≤ *t*_*n*_ ≤ *t*). Let *t*_*i*_ − *s*_*i*_ = *τ*_*i*_ and ∑*τ*_*i*_ = *τ*, where *i* = 1,2,…, *n*.

The amount of loss is the sum of the differences between the arrival and the service in the *n* loss periods. For all *i* ∈ [1,2,…, *n*], we have

(9) $$\begin{array}{c} L\left(s_{i},t_{i}\right)=A\left(s_{i},t_{i}\right)-S\left(s_{i},t_{i}\right), \end{array}$$

and the total loss during [*s*, *t*] is

(10) L(s,t)=∑i=1nL(si,ti)=∑i=1n(A(si,ti)−S(si,ti))=∑i=1n((A(si,ti)−α(τi))+(β(τi)−S(si,ti)))−∑i=1n(β(τi)−α(τi)),

where *i* ∈ [1,2,…, *n*].

Then the loss bound can be expressed as

(11) P{L(s,t)>x} =P{∑i=1n((A(si,ti)−α(τi))+(β(τi)−S(si,ti)))   −∑i=1n(β(τi)−α(τi))>x} =P{∑i=1n((A(si,ti)−α(τi))+(β(τi)−S(si,ti)))    >x+∑i=1n(β(τi)−α(τi))}.

According to the properties of ⊗ and Lemma 4, we can get the following inequality from (11):

(12) P{L(s,t)>x}  ≤(f⊗⋯⊗f︸n  occurrences⊗g⊗⋯⊗g︸n  occurrences)(x+∑i=1n(β(τi)−α(τi))) =((f⊗⋯⊗f︸n  occurrences)⊗(g⊗⋯⊗g︸n  occurrences))   ×(x+∑i=1n(β(τi)−α(τi)))  =(f⊗g)n(x+∑i=1n(β(τi)−α(τi))).

For a nonnegative wide-sense decreasing function *h*, there exists a *δ* that makes *h* ⊗ *h*(*x*) = *δh*(*x*), where *δ* > 1. Hence, (12) can be simplified as

(13) $$\begin{array}{c} \lambda \left(f⊗g\right)\left(x+\overset{n}{\underset{i=1}{\sum }}\left(\beta \left(\tau_{i}\right)-\alpha \left(\tau_{i}\right)\right)\right), \end{array}$$

where *λ* > 1.

In the long term, the arrival traffic will not exceed the service provided by the network system (or the system will collapse). Hence, for stability, we assume *β*(*x*) ≥ *α*(*x*) for all *x* ≥ 0.

Since

(14) $$\begin{array}{c} \overset{n}{\underset{i=1}{\sum }}\left(\beta \left(\tau_{i}\right)-\alpha \left(\tau_{i}\right)\right)\in \left[0,\beta \left(t-s\right)-\alpha \left(t-s\right)\right], \end{array}$$

there exists a *η* ∈ [0,1] making

(15) $$\begin{array}{c} \eta \left[\beta \left(t-s\right)-\alpha \left(t-s\right)\right]=\overset{n}{\underset{i=1}{\sum }}\left(\beta \left(\tau_{i}\right)-\alpha \left(\tau_{i}\right)\right). \end{array}$$

It is obvious that we can find a *r*, where *r* ≥ *λ* > 1 which makes

(16) r(f⊗g)(x+β(t−s)−α(t−s)) =λ(f⊗g)(x+η[β(t−s)−α(t−s)]),

since *f* ⊗ *g* is a nonnegative wide-sense decreasing function. Then the theorem is proved.

Note that *r* is determined by the arrival traffic, the service provided by the network system, and the buffer size *b* together. If *r* can be denoted by a function *R*(*A*(*s*, *t*), *S*(*s*, *t*), *b*), then (8) can be rewritten as

(17) P{L(s,t)>x} ≤R(A(s,t),S(s,t),b)f⊗g(x+β(t−s)  −α(t−s)).

When the buffer size *b* approaches to infinity or the service provided by the network *S*(*s*, *t*) is larger than or equal to the arrival traffic *A*(*s*, *t*) for all 0 ≤ *s* ≤ *t*, *R*(*A*(*s*, *t*), *S*(*s*, *t*), *b*) should be small enough making *R*(*A*(*s*, *t*), *S*(*s*, *t*), *b*)*f* ⊗ *g*(*x* + *β*(*t* − *s*) − *α*(*t* − *s*)) tend to zero.

There is one thing should be noticed that our analysis is based on t.a.c. stochastic arrival curve and stochastic strict service curve. However, there are many kinds of traffic that can-not be characterized by t.a.c. stochastic arrival curve but can be modeled by v.b.c. stochastic service curve or m.b.c. stochastic arrival curve. In some cases, only stochastic strict service curve or weak stochastic network calculus can be obtained [12]. The forms of these arrival curves and these service curves are presented in Tables 1 and 2, and their detailed definitions can be referred to in [14]. Our loss analysis model can-not be directly applied under these scenarios. Fortunately, this problem can be solved by using the model transform in stochastic network calculus. By the model transform, we can readily obtain the t.a.c. stochastic arrival curve and the stochastic strict service curve if we deduce any other types of stochastic arrival curves and stochastic service curves. The details of the model transform are presented in [18].

## 4. Loss Bounds for Multiple Input Flows

**Figure 2:**
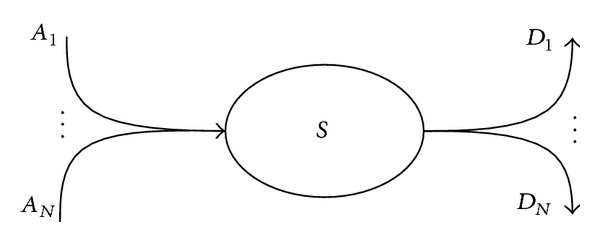
A network with multiple input flows.

Compared to deterministic network calculus, statistical multiplexing gain is a great advantage of stochastic network calculus. Hence, in this section, we consider a network scenario with multiple input flows as shown in Figure 2. We will prove that our analysis model mentioned in the previous section can be also applied to this scenario. The superposition property given by Theorem 7 is necessary for our latter analysis.

**Theorem 7 (superposition)**

Consider *N* flows with arrival processes *A*_*i*_(*t*), *i* = 1,…, *N*, respectively. Let *A*(*t*) denote the aggregate arrival process, or *A*(*t*) = ∑_*i*=1_^*N*^ *A*_*i*_(*t*). If ∀*i*, *A*_*i*_ ∼_*ta*_〈*f*_*i*_, *α*_*i*_〉, then *A* ∼_*ta*_〈*f*, *α*〉 where *f*(*x*) = *f*_1_ ⊗ ⋯⊗*f*_*N*_(*x*) and *α*(*t*) = ∑_*i*=1_^*n*^ *α*_*i*_(*t*).

**Proof**

Consider

(18) P{A(s,t)−α(t−s)>x} =P{∑i=1NAi(s,t)−∑i=1Nαi(t−s)>x} =P{∑i=1N(Ai(s,t)−αi(t−s))>x}.

According to Lemma 4, we obtain

(19) $$\begin{array}{c} P\left\{\overset{N}{\underset{i=1}{\sum }}\left(A_{i}\left(s,t\right)-\alpha_{i}\left(t-s\right)\right)>x\right\}\leq f_{1}⊗\cdots ⊗f_{N}\left(x\right). \end{array}$$

The theorem is proved by applying (19) to (18).

The loss bounds for the aggregate flow and every single flow are given by Theorem 8.

**Theorem 8**

Consider a network system with finite buffer that is fed with *N* flows with arrival processes *A*_*i*_(*t*), *i* = 1,2,…, *N*, respectively. Let *A*(*t*) denote the aggregated arrival process. If the system provides a stochastic strict service curve *S* ∼_*ssc*_〈*g*, *β*〉 and ∀*i*, *A*_*i*_ ∼_*ta*_〈*f*_*i*_, *α*_*i*_〉, then if ∑_*i*=1_^*N*^ *α*_*i*_(*t*) < *β*(*t*) for all *t* ≥ 0, the loss bounds for the aggregated flow and for each single flow *i* are

(20) $$\begin{array}{c} P\left\{L\left(s,t\right)>x\right\}\leq r\left(f⊗g\right)\left(x+\beta \left(t-s\right)-\alpha \left(t-s\right)\right), \end{array}$$

(21) $$\begin{array}{c} P\left\{L_{i}\left(s,t\right)>x\right\}\leq r_{i}\left(f⊗g\right)\left(x+\beta \left(t-s\right)-\alpha \left(t-s\right)\right), \end{array}$$

where *f*(*x*) = *f*_1_ ⊗ ⋯⊗*f*_*N*_(*x*), *α*(*t*) = ∑_*i*=1_^*N*^ *α*_*i*_(*t*), and *r*_*i*_ and *r* are some constants in (1, +*∞*).

**Proof**

According to Theorem 7, the aggregate process *A*(*t*) has a t.a.c. arrival curve *α*(*t*) = ∑_*i*=1_^*N*^ *α*_*i*_(*t*) with bounding function *f*(*x*) = *f*_1_ ⊗ ⋯⊗*f*_*N*_(*x*). Then we can readily get (20) by Theorem 6.

Let *S*_*i*_(*s*, *t*) denote the service provided to flow *i* by the network system during [*s*, *t*]; then,

(22) Si(s,t)=S(s,t)−∑j=1,j≠iNAj(s,t),P{Si(s,t)<β(t−s)−∑j=1,j≠iNαj(t−s)−x}  =P{S(s,t)−∑j=1,j≠iNAj(s,t)<β(t−s)       −∑j=1,j≠iNαj(t−s)−x}  =P{β(t−s)−S(s,t)       +∑j=1,j≠iN(Aj(s,t)−αj(t−s))>x}  ≤g⊗f1⊗⋯⊗fi−1⊗fi+1⊗⋯⊗fN(x).

So the network system provides to the flow *i* a stochastic strict service curve denoted by *S*_*i*_ ∼_ssc_〈*g*_*i*_, *β*_*i*_〉, where *β*_*i*_(*t*) = *β*(*t*) − ∑_*j*=1,*j*≠*i*_^*N*^ *α*_*j*_(*t*) and *g*_*i*_(*x*) = *g* ⊗ *f*_1_ ⊗ ⋯⊗*f*_*i*−1_ ⊗ *f*_*i*+1_ ⊗ ⋯⊗*f*_*N*_(*x*). Then the loss bound for flow *i* can be obtained from Theorem 6

(23) P{Li(s,t)>x} ≤rifi⊗gi(x+βi(t−s)−αi(t−s)) =rifi⊗g⊗f1⊗⋯⊗fi−1⊗fi+1⊗⋯  ⊗fN(x+β(t−s)−∑j=1,j≠iNαj(t−s)−αi(t−s)) =rif1⊗⋯⊗fN⊗g(x+β(t−s)−α(t−s)) =rif⊗g(x+β(t−s)−α(t−s)).

## 5. Loss Factor

In this section, we will explore how the input traffic, the service provided by the server, and the buffer size affect the value of loss factor by simulation.

### 5.1. Simulation Model

**Figure 3:**
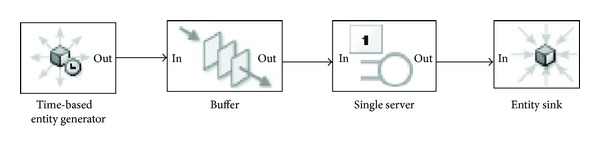
The simulation model.

The simulations were implemented in SimEvents which provides a discrete-event simulation engine and component library for Simulink. We consider the same network system as depicted in Figure 1. The corresponding simulation model should be as Figure 3 shows.

However, when the buffer block is full, the IN port of this block is unavailable and the previous block stops generating entities. That means there will be no more packets arriving at the server while the queue of the server is full, which conflicts with the cause of packet loss we assumed above. Hence, we design a revised model to accommodate to our loss analysis model.

**Figure 4:**
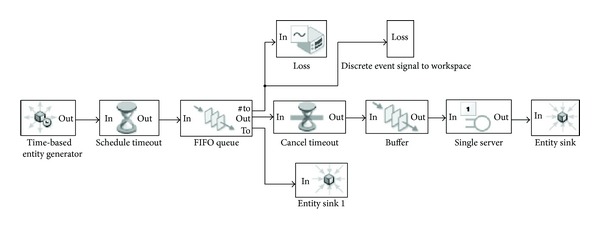
The revised simulation model.

In Figure 4, we use the schedule timeout block, the FIFO queue block, and the cancel timeout block to simulate the loss process. The schedule timeout block schedules a timeout event for each arriving entity. When the buffer block is full, the new arriving entities will be queuing in the FIFO Queue block. We set the timeout interval of the schedule timeout block for a small value. Then once there are entities in the FIFO queue block, they will be dropped in a very short time.

### 5.2. Arrival Curve and Service Curve

We assume that all packets have the same length and an entity in the simulation model denotes a packet. We assume that the server adopts FIFO and the service time for one packet is *δ*. We assume the arrival traffic follows a Poisson process with the parameter *λ*.

For a Poisson process *A*(*t*), we have

(24) $$\begin{array}{c} P\left\{A\left(s+t\right)-A\left(s\right)=n\right\}=e^{-\lambda t}\frac{{\left(\lambda t\right)}^{n}}{n!}. \end{array}$$

Then the traffic arrival curve can be expressed as

(25) P{A(s,s+t)−λt>x}=P{A(s+t)−A(s)>λt+x}≤∑n=⌈x+λt⌉∞e−λt·(λt)nn!.

The factorial and the sum make it hard to calculate the bounding function. We use an approximation result provided in [19] which gives a much simpler bounding function

(26) $$\begin{array}{c} P\left\{A\left(s,s+t\right)-\lambda t>x\right\}\leq e^{x-(\lambda t+x)\ln((\lambda t+x)/\lambda t)}. \end{array}$$

Since the service time of each packet is assumed to be a fixed value, we can readily get the service curve

(27) $$\begin{array}{c} S\left(s,s+t\right)=ct, \end{array}$$

where *c* = 1/*δ*.

According to Theorem 6, the loss bound is obtained as

(28) $$\begin{array}{c} P\left\{L\left(s,s+t\right)>x\right\}\leq rf\left(x+ct-\lambda t\right), \end{array}$$

where *f*(*x*) = *e* ^*x*−(*λt*+*x*)ln⁡⁡((*λt*+*x*)/*λt*)^.

### 5.3. Simulation Results

In this part, we will try to find out the correlations between the loss factor, buffer size, the arrival traffic and the service. Then three different sets of parameters for simulations are adopted as shown in Table 3.

The value of the loss factor can-not be directly obtained by the simulation. Because the simulation result can only provide the amount of loss packets and the result provided by (28) is the CCDF of the loss. Then we design a method to obtain an approximate value of the loss factor. For a certain parameter family, we do the simulation for 100 times and get 100 corresponding values of the amount loss packets. Let *l* denote the fiftieth largest number of these 100 values. Then we get 10 such kind of *l* for every set of experiments as shown in Table 4. For every *l*, we use the following equation to derive an approximate value of the loss factor *r*:

(29) P{L(t)>l}=rf(l+ct−λt)=rel+ct−λt−(l+ct)ln⁡⁡((l+ct)/λt)=0.5.

**Figure 5:**
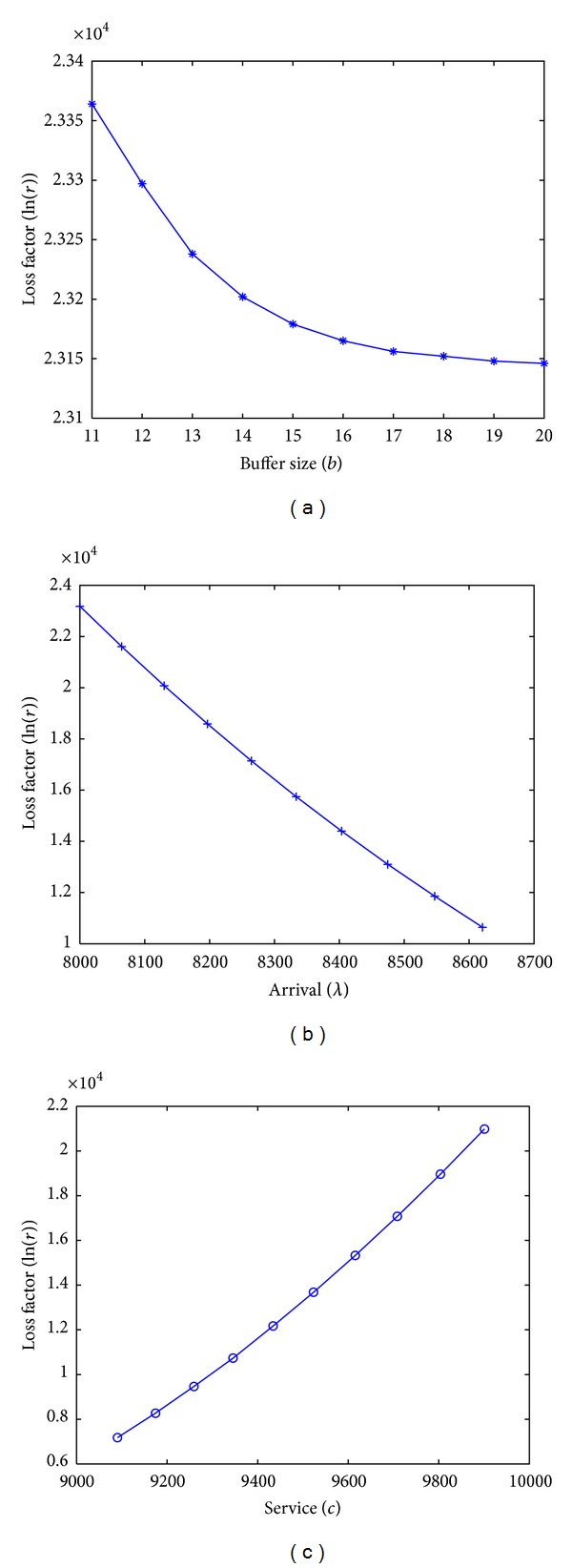
Curves between the loss factor and the buffer size, the loss factor and the arrival rate, and the loss factor and the service rate.

The data in Table 4 is reasonable that the amount of loss increases with the arrival rate and decreases with the buffer size and the service rate. Overall, if the arrival curve and the service curve are fixed, the smaller the loss factor *r*, the tighter the loss bound *L*(*s*, *t*). From (29), it is obvious that the loss factor *r* increases with *l* + *ct* − *λt*, as *f*(*x*) is a decreasing function. Hence, when the buffer size increases, the amount of loss *l* decreases; then the loss factor *r* decreases. When the arrival rate *λ* increases, *l* + *ct* − *λt* decreases; then the loss factor decreases. The relationship between the loss factor *r* and the service rate *c* can be analysed in the same way, and Figures 5(a), 5(b), and 5(c) show the corresponding curves. We can find functions to fit the curves, that means if two of the arrival, the service, and the buffer size are certain, we can adjust the other one to get a proper value of loss factor to make the loss bound at a desired level.

## 6. Conclusion

Stochastic network calculus is a very useful tool for performance analysis. However, there is a defect in stochastic network calculus that it is not easy to be used for loss analysis. In this paper, a new parameter named loss factor is proposed into stochastic network calculus. And then the loss bound based on t.a.c. stochastic arrival curve and stochastic strict service curve is derived via loss factor. We also proved that our result is valid when there are multiple flows arriving into the network.

We only analyzed the correlation between the loss factor and buffer size, the correlation between the loss factor and the arrival traffic, and the correlation between loss factor and the service, respectively, in this paper. Finding a way to denote the loss factor by the arrival cure, the service cure, and the buffer size combined remains as a challenge.

## Figures and Tables

**Table 1:** Stochastic arrival curves.

Type	Form
t.a.c.	*P*{*A*(*s*, *t*) − *α*(*t* − *s*) > *x*}
v.b.c.	*P*{sup⁡_0≤*s*≤*t*_[*A*(*s*, *t*) − *α*(*t* − *s*)] > *x*}
m.b.c.	*P*{sup⁡_0≤*s*≤*t*_sup⁡_0≤*u*≤*s*_[*A*(*u*, *s*) − *α*(*s* − *u*)] > *x*}

**Table 2:** Stochastic service curves.

Type	Form
w.s.	*P*{*A* ⊗ *β*(*t*) − A*(*t*) > *x*}
s.c.	*P*{sup⁡_0≤*s*≤*t*_[*A* ⊗ *β*(*s*) − A*(*s*)] > *x*}
s.s.c.	*P*{*S*(*s*, *t*) < *β*(*t* − *s*) − *x*} ≤ *g*(*x*)

**Table 3:** Simulation parameters.

Parameter	Set 1	Set 2	Set 3
*b*	11,12,…, 20	16	20
(1/*λ*)(10^−4^)	1.25	1.16,1.17,…, 1.25	1.25
*δ* (10^−4^)	1.00	1.00	1.01,1.02,…, 1.10
Simulation time	100	100	100

**Table 4:** Simulation Results.

*l*	1	2	3	4	5	6	7	8	9	10
Set 1	987	687	427	263	162	99	60	38	23	14
Set 2	1051	979	784	630	501	400	319	256	204	162
Set 3	20	30	44	64	90	130	184	254	358	490
